# Neoadjuvant B-RAF and MEK Inhibitor Targeted Therapy for Adult Papillary Craniopharyngiomas: A New Treatment Paradigm

**DOI:** 10.3389/fendo.2022.882381

**Published:** 2022-06-09

**Authors:** Francesco Calvanese, Timothée Jacquesson, Romain Manet, Alexandre Vasiljevic, Hélène Lasolle, Francois Ducray, Gerald Raverot, Emmanuel Jouanneau

**Affiliations:** ^1^ Pituitary and Skull Base Neurosurgical Department, Reference Center for Rare Pituitary Diseases HYPO, “Groupement Hospitalier Est” Hospices Civils de Lyon, “Claude Bernard” Lyon 1 University, Hôpital Pierre Wertheimer, Lyon, France; ^2^ Department of Neurosurgery, I.R.C.C.S. San Raffaele Scientific Institute, Vita-Salute University, Milan, Italy; ^3^ Lyon University, Université Claude Bernard Lyon 1, Lyon, France; ^4^ CREATIS Laboratory CNRS UMR5220, Inserm U1206, INSA-Lyon, University of Lyon 1, Lyon, France; ^5^ Department of Pathology, Groupement Hospitalier, Lyon, France; ^6^ INSERM U1052, CNRS UMR5286, Cancer Research Center of Lyon, Lyon, France; ^7^ INSERM U1028, CNRS UMR5292, Lyon Neuroscience Research Center, Neuro-Oncology & Neuro–Inflammation Team, Lyon, France; ^8^ Endocrinology Department, Reference Center for Rare Pituitary Diseases HYPO, “Groupement Hospitalier Est” Hospices Civils de Lyon, “Claude Bernard” Lyon 1 University, Hôpital Louis Pradel, Lyon, France; ^9^ Cancerology Research Center of Lyon, INSERM U1052, CNRS UMR 5286, Cancer Cell Plasticity Department, Transcriptome Diversity in Stem Cells Laboratory, Lyon, France; ^10^ Service of Neuro-Oncology, Hospices Civils de Lyon, Groupement Hospitalier Est, Neurology Hospital, Lyon, France

**Keywords:** papillary craniopharyngiomas, tumor biopsy, V600E BRAF mutation, B-RAF and MEK inhibitor targeted therapy, neoadjuvant treatment

## Abstract

**Background:**

Surgical and clinical management of craniopharyngiomas is associated with high long-term morbidity especially in the case of hypothalamic involvement. Improvements in knowledge of craniopharyngioma molecular biology may offer the possibility of safe and effective medical neoadjuvant treatments in a subset of patients harboring papillary subtype tumors with a BRAFV600E mutation.

**Method:**

We report herein two cases of tubero-infundibular and ventricular Papillary Craniopharyngiomas in which BRAF/MEK inhibitor combined therapy was used as adjuvant (Case 1) or neoadjuvant (Case 2) treatment, with a 90% reduction in tumor volume observed after only 5 months. In Case 2 the only surgical procedure used was a minimal invasive biopsy by the trans-ventricular neuroendoscopic approach. As a consequence, targeted therapy was administered in purely neoadjuvant fashion. After shrinkage of the tumor, both patients underwent fractionated radiotherapy on the small tumor remnant to achieve long-term tumor control. A review of a previously reported case has also been performed.

**Result:**

This approach led to tumor control with minimal long-term morbidity in both cases. No side effects or complications were reported after medical treatment and adjuvant radiotherapy.

**Conclusion:**

Our experience and a review of the literature argue for a change in the current treatment paradigm for Craniopharyngiomas (CPs). In giant and invasive tumors, confirmation of BRAFV600E mutated PCPs by biopsy and BRAF/MEK inhibitor therapy before proposing other treatments may be useful to improve long term outcomes for patients.

## Introduction

Craniopharyngiomas (CPs) are rare suprasellar tumors arising from the epithelium of craniopharyngeal duct remnants with a global incidence of 0.5-2.5 new cases per 1 million population (1, 2). They develop along the hypothalamic-pituitary axis and exhibit two distinct histological subtypes: Adamantinomatous (ACPs) and Papillary (PCPs) craniopharyngiomas. ACPs account for 90% of all and present a bimodal peak of incidence in childhood and in adulthood whereas, PCP represents 10% of all craniopharyngiomas and usually affect adult patient in 4^th^-5^th^ decade of life (1–3).

Despite CPs being classified as low-grade neoplasms (Grade I, WHO), they show an aggressive local behavior and a high rate of recurrence (i.e., from 9 to 62%), requiring multimodal invasive treatments to achieve tumor control (1, 2, 4–6). The involvement of the third ventricle is a critical factor increasing long-term morbidity and limiting the effectiveness of surgery and/or radiotherapy (5, 7–11). Pascal and Prieto (8, 12, 13) classified CPs topographically into four categories based on their relationship with third ventricular floor: Suprasellar (SS) or pseudo-intraventricular, SS secondary intraventricular, infundibular-tuberal or not strictly intraventricular and ‘‘purely” intra-ventricular tumors. The surgical resection of intraventricular and/or giant CPs is particularly challenging due to the frequent third ventricular floor invasion and narrow surgical corridors (4, 9, 12–14). Although in some cases the third ventricle portion can be safely resected, ventricular remnants are frequent after surgery, require adjuvant radiotherapy, and increase the risk of long-term recurrence and morbidity (10).

Improving our knowledge of the genetic landscape of craniopharyngiomas has led to characterization of two different clonal driver mutations that control oncogenesis of the two histological subtypes (3). ACPs are characterized by alterations in the Wnt/β-catenin pathway, mainly involving the central regulatory gene CTNNB1, whereas most PCPs are driven by the V600E mutation in the BRAF gene, which activates the mitogen-activated protein kinase (MAPK) signaling pathway (3, 5, 15, 16). These molecular changes have revealed potential targets for new therapeutics that could improve long term control of tumor volume with less morbidity (6, 16).

To date, no target agents have been found to have efficacy in blocking Wnt/B-catenin pathway in ACPs (1, 3, 16). Nonetheless, target therapy with B-RAF and MEK inhibitor agents has shown good results in the treatment of a number of human cancers (6, 16–18) and glial tumors (16, 18, 19) harboring the V600E BRAF mutation. These results have led to successful use of these agents for aggressive PCPs that present with a high frequency of BRAF V600E mutation (3, 16–21).

We present herein two cases that showed efficacy of combined anti-BRAF/MEK therapy as adjuvant and neoadjuvant treatment of a PCP. In view of our results and a review of the literature we then discuss a new concept for the management of invasive CPs.

## Materials and Methods

**Figure 1:**
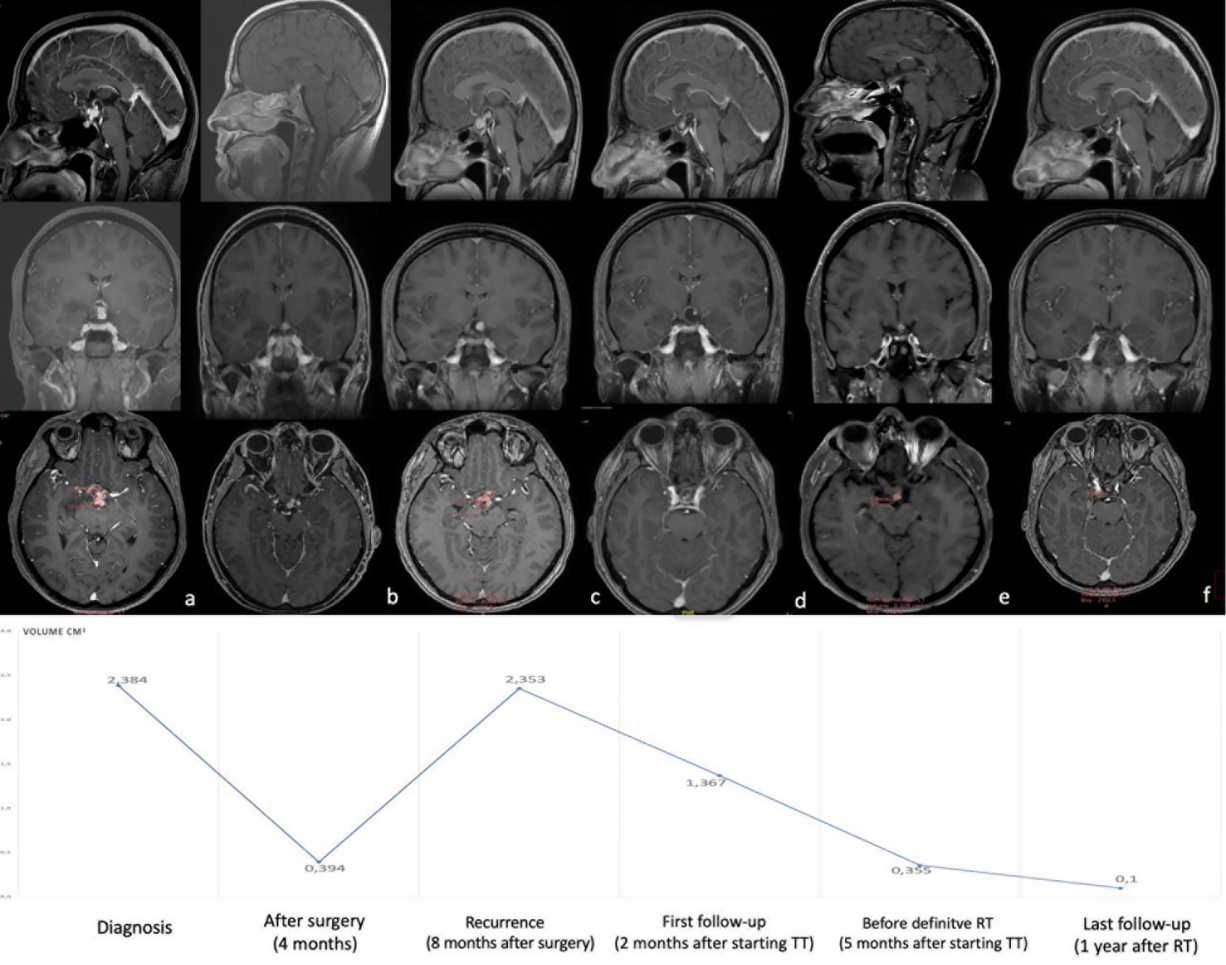
Post-gadolinium axial, coronal and sagittal T1WI MRI images, representing the clinical course in case 1. **(A, B)** Shows the tumor volume and presentation at time of diagnosis (25.4 x 15.0 mm maximal axis and 2.384 cm3 volume) and after surgery (maximal axis: 7.5 x 11.5 mm, volume: 0.394 cm3). **(C)** Shows tumor recurrence/regrowth at 12 months postoperatively (13 x 24 mm and a volume of 2.353 cm3). **(D, E)** Show dramatic and rapid reduction in tumor volume at 2 months (80 %) and 4 months (90%) after starting combined anti-BRAF/MEK therapy. **(F)** Shows results at 1 year after final radiotherapy (near complete response). Volume curve has been reported in the inferior part of figure. TT: B-RAF and MEK inhibitor targeted therapy; RT: radiotherapy.

**Figure 2:**
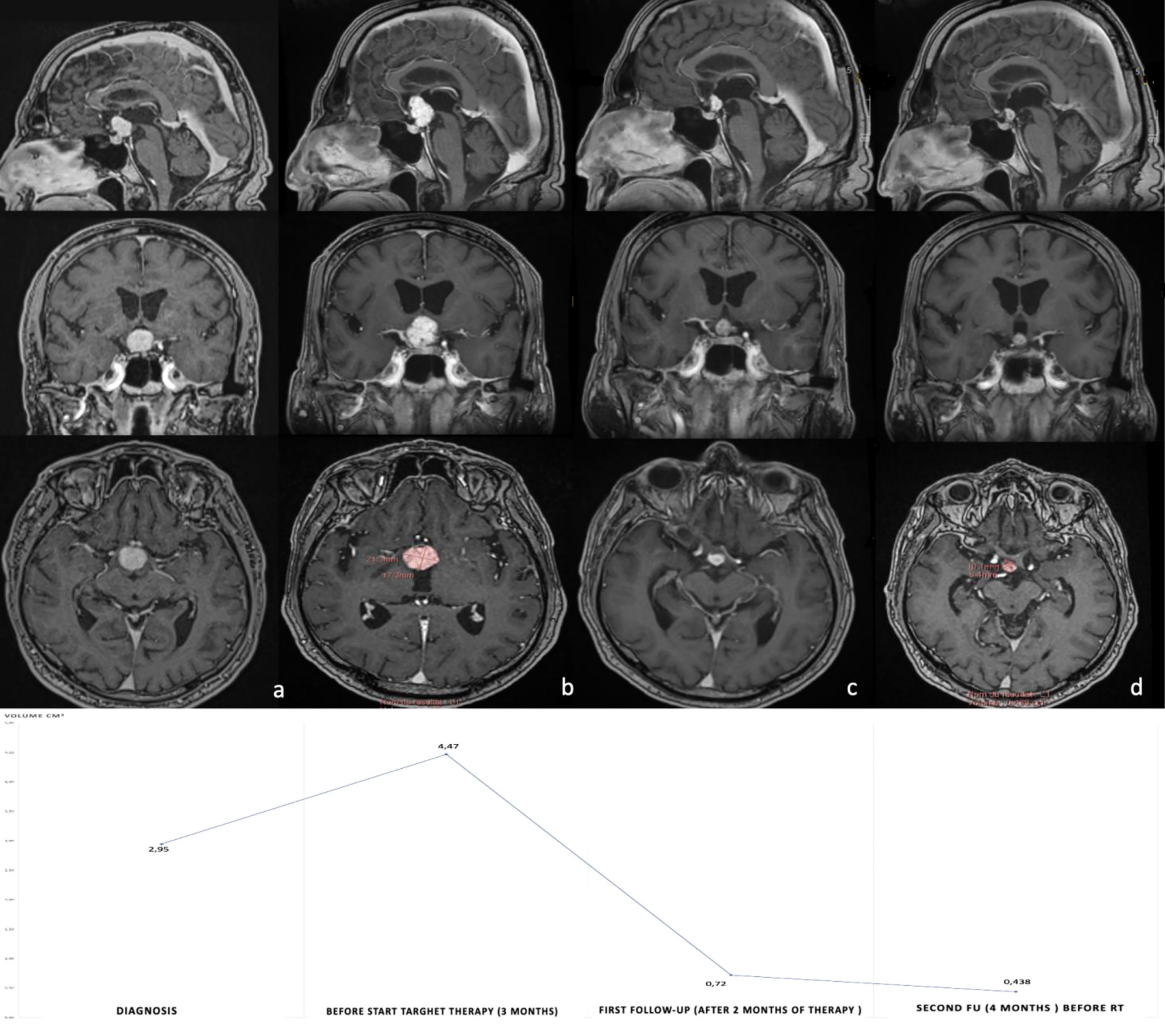
Post-gadolinium axial, coronal and sagittal T1WI MRI images of case 2. **(A)** Postcontrast T1-weighted image shows large homogeneously enhanced intraventricular mass measuring 19 x 18.5 mm maximal axis and 2.945 cm^3^ volume. **(B)** Shows progression of the intraventricular tumor portion after trans-ventricular endoscopic biopsy (18% of tumor volume). Panels c and d show a dramatic reduction in volume at 2 months **(C)** and 4 months **(D)** after commencing combined BRAF/MEK inhibitor treatment. Note complete resolution of the mass effect on suprasellar neurovascular structures and on Monro’s foramen. Volume curve has been reported in the inferior part of figure. RT, radiotherapy.

Two patients were treated for ventriculo-tuberal complex PCP with adjuvant (Case 1) and neoadjuvant (Case 2) anti-BRAF/MEK therapy at the Pierre Wertheimer Neurological Institute between 2019 and 2021. Preoperative, postoperative and follow-up radiological, biochemical, and clinical findings for both patients were collected and are reported in the results section and in **Figures 1**, **2**. In accordance with our institutional policy, both patients gave their informed consent for surgical operations, medical treatment and radiotherapy and for the use of their clinical data for research and publication purposes.

A systematic review of the pertinent medical literature was performed within PUBMED and Google scholar databases. All research which included the following keywords: ‘‘Papillary craniopharyngioma’’, ‘‘Papillary craniopharyngioma AND medical treatment’’, ‘‘Papillary craniopharyngioma AND BRAF inhibitors’’ and ‘‘Papillary craniopharyngioma AND BRAF/MEK inhibitors’’ was reviewed. Using the bibliographies of articles identified in our primary search we then performed a secondary search. Articles were reviewed by title and abstract for potential relevance as well as being reviewed completely if the title or/and abstract did not clearly indicate the degree of relevance. The search was limited to human subjects and English language publications. Only full papers and relevant publications as well as original communications were selected.

## Results

### Case Reports

#### CASE 1

A 40-year-old man was admitted to our institution with a 2-month history of bitemporal inferior quadraniopsia and a decrease in right visual acuity confirmed by ophthalmological evaluation. Cerebral MRI showed a tuberoinfundibular solid-cystic mass, infiltrating the third ventricular floor and measuring 25.4 x 15.0 mm maximal axis and 2.384 cm^3^ in volume (**Figure 1A**). The lesion showed heterogenous contrast enhancement after gadolinium on T1WI and hyperintensity on T2WI without intra-tumoral calcification on CT scan. Endocrinological pituitary screening showed a central hypogonadism without other deficits nor diabetes insipidus.

The patient underwent a near-total resection through an extended trans tubercular transsphenoidal endoscopic approach. Post-operative cerebral MRI showed tumor volume reduction of 90% (maximal axis: 7.5 x 11.5 mm, volume: 0.394 cm^3^) without posterior III floor hypothalamic damage (**Figure 1B**). The post-operative course showed a complete regression of visual symptoms but the patient developed diabetes insipidus and central hypothyroidism requiring substitutive treatment. The patient was discharged from hospital after seven days. Histopathological analysis demonstrated a papillary Craniopharyngioma harboring the BRAF V600E mutation. The first MRI at 4 months showed stable disease but the second MRI, 8 months post-surgery, demonstrated tumor growth and there was new visual impairment. The lesion showed a maximal axis of 13 x 24 mm and a volume of 2.353 cm^3^ (94% increase in tumor volume, **Figure 1C**). After discussion within our Multidisciplinary Pituitary Tumor Board, the patient commenced target therapy with Dobrafenib (150 mg Twice daily) and Trametinb (2 mg once daily) for 5 months. Indeed, in view of both hypothalamus and chiasma infiltration and the rapidity of recurrence, a second surgery was excluded, and radiotherapy was delayed in the hope of having a smaller target. The first follow-up cerebral MRI, performed 2 months after start of treatment, showed a 40% reduction in tumor volume. (1.367 cm^3^) (**Figure 1D**). Ophthalmologic examination showed a normal result. Combined treatment was continued and well-tolerated without side effects. Cerebral MRI performed at 5 months post-treatment showed a 90% reduction in tumor volume (0.355 cm^3^). (**Figure 1E**). Subsequently, fractionated VMAT (Volumetric Modulated Arc Therapy) radiotherapy with a total dose of 52.2 Gy in 29 fractions was applied, while combined treatment was interrupted one month before radiotherapy to prevent radio-sensitization. At last follow up, one year after radiotherapy, the tumor showed a “near complete” radiological response, the patient was symptom-free and had resumed normal life (**Figure 1F**).

#### CASE 2

A 69-year-old HIV-seropositive man was referred to our center after a one year history of frontal headaches, a right visual impairment and psychiatric changes (aggressivity and behavior changes). Cerebral MRI revealed a large solid third ventricular lesion measuring 19 x18.5 mm in maximal axis and 2.945 cm^3^ in volume (**Figure 2A**). The lesion was implanted on the infundibular recess and bilaterally reached Monro’s foramen. The lesion showed non-homogeneous contrast enhancement and was hypointense on T1WI and hyperintense on T2WI MRI. Hormonal screening showed normal pituitary function except for a slight disconnection hyperprolactinemia and no evidence of diabetes insipidus. Ophthalmologic evaluation revealed a left optic atrophy but visual field and acuity were normal. A biopsy by a trans-ventricular neuroendoscopic approach was performed in order to confirm the diagnosis and exclude differential diagnoses such as primary cerebral lymphoma. Histopathological analysis showed a papillary craniopharyngioma harboring classical BRAF V600E mutation.

After discussion within our Multidisciplinary Pituitary board, considering the invasion of the hypothalamus, neoadjuvant targeted therapy treatment was decided. A combination of dobrafenib (150 mg Twice daily) and Trametinb (2 mg once daily), after optimization of antiviral drug to avoid pharmacokinetic interactions, was started. MRI performed 3 months after the diagnosis and before starting targeted therapy showed tumor progression with a volume of 4.469 cm^3^ and maximal axis of 21.3 x17.3 mm (an increase of 18% in tumor volume) (**Figure 2B**). After two months of therapy, the patient showed a complete regression of visual dysfunction and an improvement in psychiatric symptoms. Treatment was well-tolerated without side effects. At that date, MRI showed a near total response with an 80% reduction in tumor volume (0.72 cm^3^) (**Figure 2C**) associated with complete resolution of the Monro’s foramen obstruction. The tumor volume continued to diminish with 4 month follow-up imaging showing a total volume reduction of 90% (0.438 cm^3^) (**Figure 2D**), allowing us to perform fractionated radiation treatment as initially planned. Fractionated Radiation therapy (52 Gy/30 Fraction) was scheduled 6 months after treatment initiation and targeted therapy was stopped 2 weeks before the start of radiation.

### Literature Review

Our primary search identified 170 papers. Twenty-two articles were selected for clinical and subject relevance. Only 11 previously reported cases of PCP treated by targeted therapy were found in the English language publications (16, 17, 19, 22–29) and a summary of these findings is shown in **Table 1**. The preliminary data of one randomized study, which analyzed adjuvant anti-BRAF/MEK inhibitor therapy for PCP, have been published and are discussed in the following section (30).

**Table 1:** Literary review of all PCPs reported case treated with BRAF/MEK inhibitor agents.

Author, Year	Sex, age (year)	Previous treatments	Symptoms before target therapy	B-RAFi-Meki treatments	Duration of treatments(month)	Response (% volume reduction)	Symptoms relive	Recurrence (solid/cystic), time of recurrence	Definitive treatments for residual or recurrence	Adverse effect	Follow-up (months)
Aylwin et al, 2015 (17)	F, 27	Surgery: STRc (EEA) x2 - RT- Surgery: STRc (EEA)	VS (temporal hemianopia, LE 6/60)	Vemurafenib 960 mg BID	3	NCR (95%)	Yes (LE 6/24)	Yes (solid), 6-week	re-started vemurafenib	CSF-leak/meningitis	7
Bastianos PK. et al, 2015 (24)	M,39	Multiple Surgery: STRc (TCA x3/EEA x1)	ICHTsymptoms	Dabrafenib 150 mg BID-trametinib 2 mg BID	1.25 (38 days)	PR (81% solid part; 85% Cystic part)	Yes	No	Surgery (EEA)-RT	Low grade Fever (1 day)	18
Roque & Odia, 2016 (29)	F,47	Surgery: PRc (TCA x 1)-Ommaya - RT	H/A, left hemiparesis, behavior changes	dabrafenib 150 mg BID- trametinib 2 mg orally UID	7	PR (80%)	Yes	no	no	intermittent fever	7
Rostami et al, 2017 (26)	M,65	Surgery: STRc (EEAx1)	VS	dabrafenib 150 mg BID After 3 weeks trametinib 2 mg UID was added	3.5	NCR (91%)*	Yes	no	no	Pyrexia needing treatments interruption	2
Juratli et al, 2019 (28)	M,21	Surgery: PR(TCA)	H/A, ICHT, PI	dabrafenib 150 mg BID -trametinib 2 mg UID	6	PR (80%-90%)	Yes	no	no	NR	18
Himes et al, 2019 (22)	M,47	Surgery: STRc -RT	VS, DI	Dabrafenib 150 mg BID, after 150 mg UID, finally 225 mg BID	9	CR (>95%)	Yes	Yes (Cystic), 2 months	Medical treatment **	NR	24
Rao et al, 2019 (25)	M,35	Surgery: STRc (TCA x1)	Hy-Cognitive dysfunction	Dabrafenib 150 mg BID	24	PR (-)	Yes	Yes	NO	NR	28
Bernstein et al, 2019 (19)	M,60	Surgery : STRc x4 -RT	_	dabrafenib 150 mg BID- trametinib 2 mg UID	_	CR (-)	Yes	No	NO	Widespread verrucal keratoses	28
Distefano et al., 2020 (27)	F, 55	Surgery: STRc (EEA x1)	VS, PI	dabrafenib 150 mg BID trametinib 2 mg UID	5	NCR (95%)	Yes	Yes, (13% cystic increasing volume)	PBRT (52.2Gy/29Frz)	grade 1 fatigue (CTCAE v4.0), coughing, and peripheral edema	4.5
Khaddour, 2020 (23)	F,39	Surgery: STRc (EEA x 2)	H/A, VS	dabrafenib 150 mg BID trametinib 2 mg UID	9	PR (70%)	yes	No	SRS-GK (25Gy isodose 50%/5frz) ***	Grade I pyrexia	9
Sabeehur Rehman Butt et al., 2021 (16)	F,32	Surgery (x1)-SRS-GK-Surgery (EEA)	–	dabrafenib 150 mg BID trametinib 2 mg UID	3	–	–	no	no	–	3
**Present Case 1**	M,	Surgery: NCRc (EEA x1)	VS, H/A	dabrafenib 150 mg BID trametinib 2 mg UID	5	NCR, 90	Yes	no	RT	No	24
**Present Case 2**	M,	No definitive treatments(Biopsy)	VS, Psychiatric disorders	dabrafenib 150 mg BID trametinib 2 mg UID	4	NCR, 90	Yes	no	RT	No	6

Note that the only case treated in pure neoadjuvant manner with medical therapy is our case 2. The reviewed results are treated in the discussion.

EEA, Endoscopic endonasal approach; STRc, subtotal resection; PRc, Partial resection; NCRc, near complete resection; NCR, Near complete response (85-95%); PR, Partial response (<80%); CR, complete response (>95%); VS, Visual symptoms or deterioration; H/A, Headache; PI, panhypopituitarism; DI, diabetes insipidus; Hy, Hydrocephalus; ICHT, symptoms of intracranial hypertension; UID, once daily; BID, twice daily.

*The rate and the magnitude of tumor volume reduction (from 11% VS 91%) significatively improved after joint administration of MEK inhibitor (trametinib).

**Probably pseudoprogression phenomenon after 3 year of radiation therapy.

***CR after SRS-GK.

Two specific surgical series were identified (31, 32). The other studies were earlier literature reviews on related topics (1–3, 5–7, 15, 18, 33).

## Discussion

Harvey Cushing referred to craniopharyngiomas as “the most forbidding of the intracranial tumors” (5). Despite improvements in microsurgical and endoscopic techniques, as well as in radiation therapy and radiosurgery, the long-term morbidity of CPs remains high, conferring a sometimes poor quality of life on these patients (1, 2, 10, 11, 14). The long-term morbidity of such tumors is mainly related to hypothalamic damage resulting either from the tumor invading neural structures or by treatment-related injury (10, 14). Consequently, craniopharyngiomas that involve the third ventricle and tuberoinfundibular areas represent the lesions which are extremely difficult to excise surgically and their subsequent management is equally difficult due to their intimate anatomical and functional relationships with the hypothalamus (9, 13, 14). According to the MRI classification proposed by Prieto et al. (8, 12), the surgical approach should be selected based on the relation of the tumor with the third ventricle floor and the value of the brainstem-mammillary body angle. In a preoperative setting, these findings must be carefully assessed to choose the best surgical approach (endonasal versus cranial) in order to reduce the aggressiveness of the surgery by avoiding, when possible, crossing the third ventricle floor or removing the hypothalamic walls (8, 10). Purely intraventricular CPs are tumors where not only the hypothalamic but also the pituitary functions can be preserved with adequate approach. However, in the case of large infundibulo-tuberal or ventricular tumors which frequently show invasion of hypothalamic structures, resection must be incomplete to avoid very serious adverse outcomes (13).

Even though PCPs account for 10% of all craniopharyngiomas in adults, they show a tendency to arise at the level of third ventricle floor and in the tuberoinfundibular area (75-90% of cases) with frequent hypothalamic involvement (1, 2, 5). This justifies their frequent presentation with hypothalamic symptoms including neuropsychiatric disorders, neurocognitive impairment and also neuroendocrine dysfunction (14, 16). Thus, complex PCPs represent a perfect example in which an effective neoadjuvant medical therapy, producing tumor shrinkage, could provide a reduction in long-term morbidity and facilitate both surgery and radiotherapy (5, 7, 21).

Brastianos et al. (20) reported in their original genetic study that PCPs harbor BRAF V600E mutation in 94.4% of cases and no other recurrent mutation or genomic alterations have been since identified (3, 20). B-RAF is an upstream regulator of the MAPK pathway which controls the cell cycle and cell proliferation (1, 16, 19, 23). BRAF V600E mutation encodes a constitutively activated B-RAF serine/threonine kinase that leads to a chronic hyperactivation of the RAS/RAF/MEK/ERK signaling pathway, driving oncogenesis in about 7% of human cancers (3, 15–18). In PCPs the mechanism by which BRAFV600E mutation is oncogenic has not yet been fully understood but it may give both a proliferative advantage to tumor SOX2+ stem cells and impair their differentiation potential (1, 15, 16).

Since the pioneering cases reported by Alwys at al. (17). and Brastianos et al. (24) in 2015, other authors have reported significant reductions in tumor volume and clinical improvement after administration of a single-agent BRAF inhibitor (17, 22, 25, 26), or combined BRAF/MEK inhibitor therapy (19, 23, 24, 26–29), in PCPs harboring the BRAF V600E mutation. The results of all previously published cases are summarized in **Table 1**. Our literature review identified 11 previous case reports. The mean reduction in tumor volume after targeted therapy was 89.2% (range 70-95%) with a minimal treatment period of 5 months (range 1.25-24 months). The most frequent adverse effect reported was low grade fever, which required brief discontinuation of treatment (17, 28). Although all of the patients in previously published reports responded to treatment, it could be argued that there was a selection bias because cases of non-responders may not have been published. Combined therapy using BRAF and MEK inhibitor seems to show a greater efficacy in the magnitude of reduction in tumor volume and in terms of rapidity of action compared to single-agent treatment (5, 16, 18, 19, 23, 26, 27). Moreover, in comparison to single-agent administration, a reduction in recurrence rate has also been described after combined therapy (19, 23, 27, 28). At the molecular level, the combination of BRAF inhibitors with MEK inhibitors could have an additive effect, augmenting the blockade of the downstream pathway of mitogen-activated protein kinase signaling (18, 23). Bernstein et al. (19) have also noted both mitigation of cutaneous toxicity and a reduction in development of resistance in those patients treated with combined therapy. Many questions remain unresolved including how long patients can be treated, how long treatment will control the tumor volume when targeted therapy is used at the time of recurrence as the only alternative treatment, as well as the long-term tolerance of such treatment (15, 16).

Although the clinical efficacy of BRAF/MEK inhibitor agents has been shown in treatment of PCPs, all previously published reports described its administration in settings of tumor recurrence or as adjuvant therapy (16). Recently, DiStefano et al. (27) and Khaddour et al. (23) reported a near complete response or a reduction of tumor volume, in 94% and 70% respectively after combined treatment with drabafenib and trametinib, followed by adjuvant radiosurgery and radiotherapy, in two patients that had rapid recurrence after partial endoscopic transsphenoidal resection. This approach is similar to that described in the present CASE 1 patient, and confirm the efficacy, rapid action and safety of combined BRAF/MEK inhibitor therapy for tumor debulking at the time of recurrence before repeat surgery or radiotherapy. Juratli et al. (28), reported using the same adjuvant approach after a partial tumor removal with severe complications (ischemia of anterior choroid artery territory and pan-hypopituitarism). Their results confirm the utility of targeted therapy in an adjuvant setting, in the case of a tumor growing after partial resection.

In view of the results achieved in case 1 in our study and the surgical risks of morbidity in giant infundibulotuberal tumors, neoadjuvant treatment was decided in our second case with a goal of minimizing hypothalamic damage. We performed a simple surgical procedure *via* a trans-ventricular neuroendoscopic approach with the sole aim of obtaining a tissue sample for histopathological and molecular analysis. Combined therapy with BRAF/MEK inhibitors administered thereafter showed rapid results, with a dramatic reduction in tumor volume of 90% at 4 months, associated with symptom relief. These results suggest its potential indication as first line treatment before surgery or radiation therapy (16, 19, 23, 27, 28). Recently, results from the ongoing phase-2 Alliance clinical trial (30), started in 2017 (NCT03224767), confirmed the high rate of volumetric response (i.e. the primary endpoint), in 15 of 16 newly patients with pathology-confirmed papillary CPs that received 1 or more cycles of combined therapy with vemurafenib and cometinib after surgery. The responders were maintained on this treatment with minimal side effects and without any additional therapy. Three patients progressed when the treatment was discontinued. This approach is different from our final proposed approach.

Despite the clinical and radiological algorithm to identify BRAF-mutated PCP that has been proposed by Fujito et al. (7), taking a tissue sample for immunohistochemistry (using the VE1 antibody) and allele-specific genetic testing remain the gold standard for identification of BRAF V600E as well as for the exclusion of the adamantinomatous subtype (1, 3, 5, 16, 31–34). Brastianos et al. also reported the presence of detectable circulating cells carrying the BRAF V600E mutation in their patient samples, but only after surgery (24). Future studies are required to confirm the validity of looking for BRAF V600E mutation in peripheral blood (liquid biopsy) prior to surgery (which may mobilize tumor cells into the general circulation). Currently, a tissue biopsy for definitive diagnosis is mandatory and can be safely performed using stereotaxic or trans-ventricular neuroendoscopic techniques, as well as *via* trans-sphenoidal endoscopic techniques (21, 31–36). Regardless of the technique used, a simple biopsy is definitely less aggressive than extended surgical resection.

**Figure 3:**
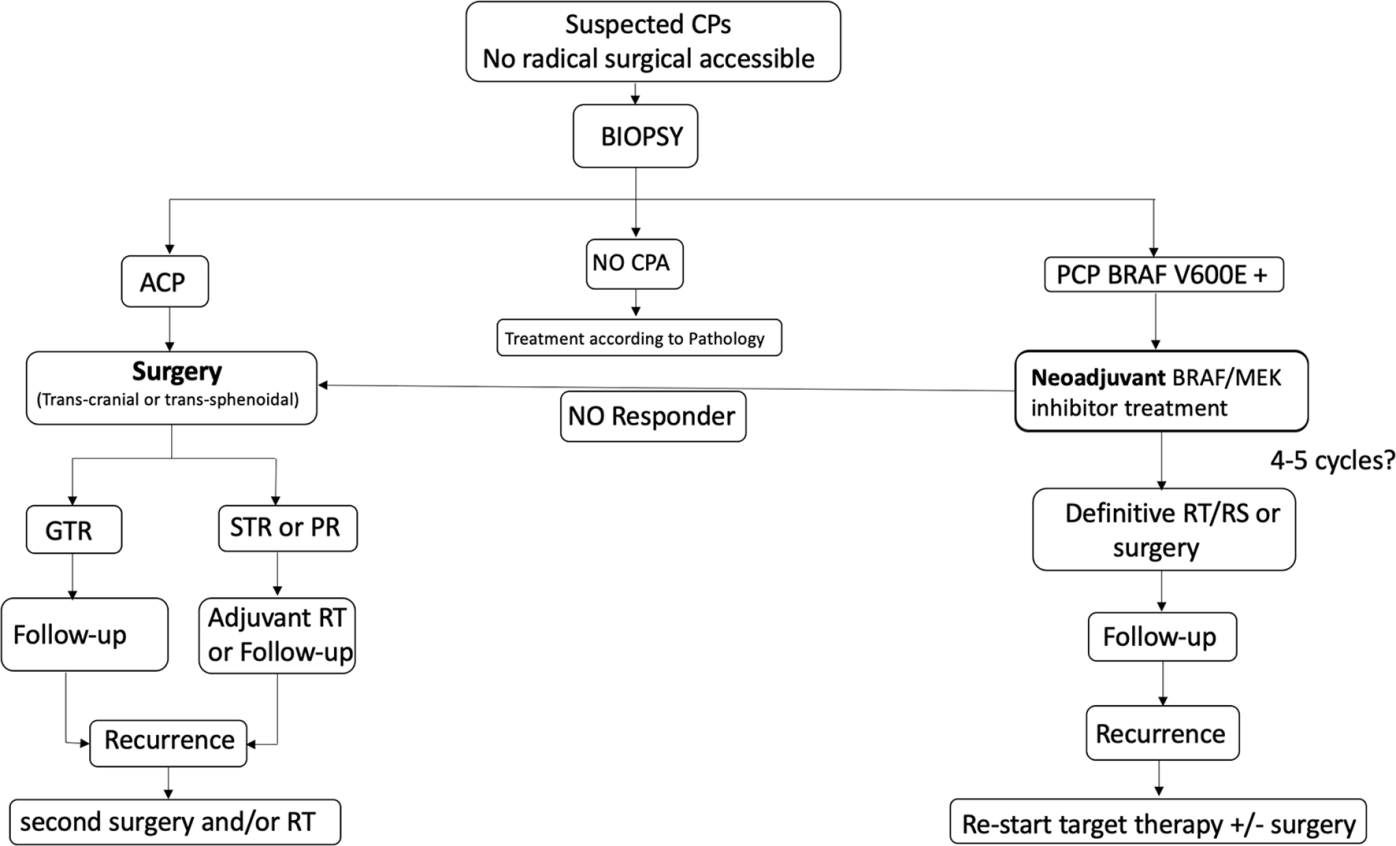
Proposed management algorithm in case of Ventricular and Infundibulo-tuberal CPA which are not good candidate for a safe radical resection. CPs, Craniopharyngioma; ACP, Adamantinomatous CPA; PCP, Papillary CPs; RT, radiotherapy; RS, radiosurgery.

In view of our experience and the above-mentioned preliminary data, a new treatment paradigm for giant and invasive craniopharyngiomas could be proposed in the hope of improving long-term patient outcomes (**Figure 3**). In these cases, a tissue biopsy should be the first option prior to making clinical decisions, even in the case of visual impairment, considering the rapid and impressive results in reducing tumor volume that are offered by medical treatment in papillary subtype tumors. In such tumors, neoadjuvant combined therapy should be applied for a few months in order to shrink the tumor before then considering a curative approach (surgery or radiotherapy/radiosurgery). Moreover, in case of rare ‘‘Purely’’ intraventicular tumors not only hypotalamic but also pituitary function could be preserved.

**Figure 4:**
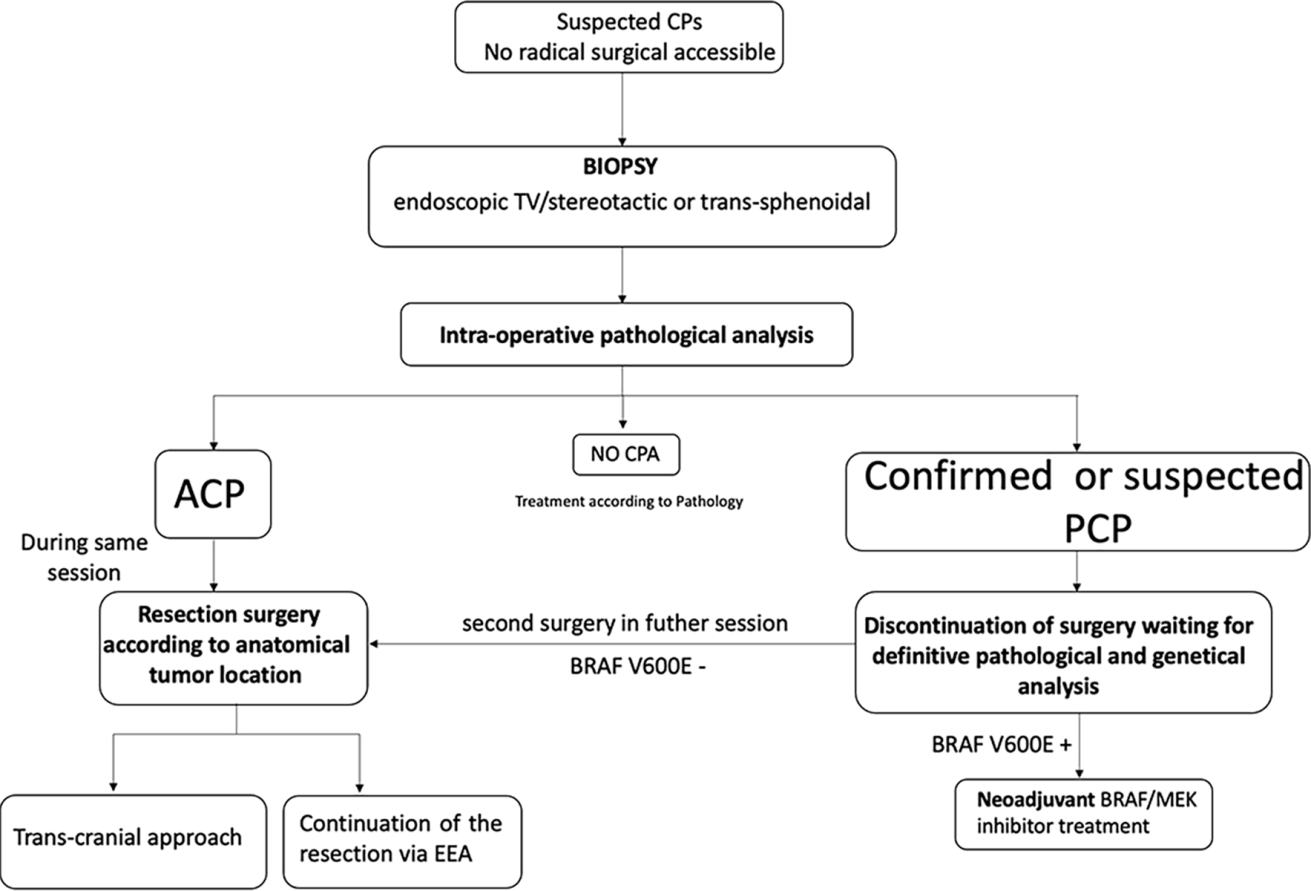
Proposed management algorithm for introperative decision making using pathological analysis on frozen section. Depending on tumor anatomical location, the biopsy can be performed either by stereotactic and neuroendoscopic transventricular techniques or using an endonasal route. In the first case, there will be two surgeries (i.e. biopsy followed by craniotomy for resection if needed) that can be done during the same anesthesia. Conversely, using endoscopic endonasal approach the biopsy could be the first step of the same procedure. See text for more details. CPs, Craniopharyngioma; ACP, Adamantinomatous CPA; PCP, Papillary CPs; EEA, endonasal endoscopic approach; TV, trans-ventricular.

The tumor biopsy could be performed using stereotactic or neuroendoscopic transventricular techniques or using an endonasal route according to tumor anatomy. In all cases, an intraoperative pathological evaluation of the tissue sample on frozen section may provide guidance for surgical decisions. In case of PCP or unconclusive result, the surgery may be discontinued waiting for definitive conclusions. In case of ACP, the procedure continues avoiding a second surgery or anesthesia for the patients. However, considering the morbidity of hypothalamic surgery, even a two-step surgery may be arguable in very huge CPs. Molecular detection of BRAF V600E mutation cannot yet be achieved in the prescribed time for intraoperative consultation (20-30 minutes). Rapid direct immunohistochemical methods are feasible but no study has tested the BRAF V600E antibody in the setting of craniopharyngioma intraoperative diagnosis (37–39). **Figure 4** reports an algorithm proposed for intraoperative decision making.

Although successfully treated BRAF-mutated CPs have similarly been described in children (40, 41), ACPs still account for the vast majority of CPs in the pediatric population. However as PCPs may exceptionally be encountered in children, the same attitude as in adult should be applied for pediatric giant CPs. Several studies are ongoing looking for possible drug targets in the adamantinomatous subtype (1, 16).

“Wait and see” management after tumor shrinkage and symptom relief can be supported by deferring radiotherapy if the lesion recurs. However, discontinuation of medical treatment after a partial or near-complete response in PCPs could be associated with a risk of early and long-term relapse due to the absence of a proven curative effect (15, 17, 18, 22, 25, 30). At the same time, long-term administration of BRAF/MEK inhibitors may increase the risk of epidermal cancer and have other side effects (18, 30). Himes et al. and Aylwin et al. reported early tumor recurrence 1-2 month after cessation of treatment (17, 22). Conversely, other authors have reported long-term tumor control after administration of radiotherapy (24, 27) or radiosurgery (23) immediately after responding to targeted therapy. Therefore, it seems reasonable to assign such treated patients to early definitive treatment to achieve long-term tumor control and to avoid tumor relapse and its hypothalamic and visual morbidity, as well as the need to resume medical therapy with unpredictable success. Moreover, tumor debulking could drastically reduce morbidity associated with surgery and radiotherapy. Although the total radiation dose is the same due to the intrinsic radiosensitivity of the tumor, the radiation field after tumor shrinkage is smaller thereby reducing the marginal dose to nearby critical structures. Likewise, shrinking tumor volume may allow radiosurgery to be used on small tumor remnants. Finally, in our opinion, definitive treatment should be provided early after a partial or near complete response to BRAF/MEK inhibitors and adapted to the anatomical location and volume of the tumor remnants as well as their surgical accessibility.

Obviously, larger prospective multicenter randomized studies are now warranted to confirm the safety and efficacy of this strategy.

## Conclusion

Changes in the algorithm for the management of craniopharyngiomas should be considered in light of progress made in molecular biology and targeted therapies. Surgery and radiotherapy remain the definitive treatments to obtain tumor control. However, a simple biopsy prior to submitting the patient to a high-risk procedure should be considered to identify a subset of patients with papillary craniopharyngiomas with BRAF mutation. This may lead to the use of a neoadjuvant targeted therapy before considering curative treatments on the smaller target. Obviously, a large cohort study is now mandatory to validate the efficacy of this new protocol. It is hoped that these drugs may decrease morbidity and improve outcomes and quality of life in patients with these tumors that have historically been surgically difficult.

## Data Availability Statement

The original contributions presented in the study are included in the article/supplementary material. Further inquiries can be directed to the corresponding author.

## Ethics Statement

Ethical review and approval was not required for the study on human participants in accordance with the local legislation and institutional requirements. The patients/participants provided their written informed consent to participate in this study. Written informed consent was obtained from the individual(s) for the publication of any potentially identifiable images or data included in this article.

## Author Contributions

Concept and design: EJ, GR, and FC. Acquisition of data: FC. Analysis and interpretation of data: All authors. Drafting the article: FC and EJ. Critically revising the article: All Authors. All authors contributed to the article and approved the submitted version.

## Conflict of Interest

The authors declare that the research was conducted in the absence of any commercial or financial relationships that could be construed as a potential conflict of interest.

## Publisher’s Note

All claims expressed in this article are solely those of the authors and do not necessarily represent those of their affiliated organizations, or those of the publisher, the editors and the reviewers. Any product that may be evaluated in this article, or claim that may be made by its manufacturer, is not guaranteed or endorsed by the publisher.
